# Molecular Systematics of the Deep-Sea Hydrothermal Vent Endemic Brachyuran Family Bythograeidae: A Comparison of Three Bayesian Species Tree Methods

**DOI:** 10.1371/journal.pone.0032066

**Published:** 2012-03-05

**Authors:** Mariana Mateos, Luis A. Hurtado, Carlos A. Santamaria, Vincent Leignel, Danièle Guinot

**Affiliations:** 1 Department of Wildlife and Fisheries, Texas A&M University, College Station, Texas, United States of America; 2 Laboratoire Mer-Molécules-Santé, Université du Maine, L'Université Nantes Angers Le Mans (L'UNAM), Le Mans, France; 3 Département Milieux et Peuplements aquatiques, Muséum National d'Histoire Naturelle, Paris, France; University of Canterbury, New Zealand

## Abstract

Brachyuran crabs of the family Bythograeidae are endemic to deep-sea hydrothermal vents and represent one of the most successful groups of macroinvertebrates that have colonized this extreme environment. Occurring worldwide, the family includes six genera (*Allograea*, *Austinograea*, *Bythograea*, *Cyanagraea*, *Gandalfus*, and *Segonzacia*) and fourteen formally described species. To investigate their evolutionary relationships, we conducted Maximum Likelihood and Bayesian molecular phylogenetic analyses, based on DNA sequences from fragments of three mitochondrial genes (16S rDNA, Cytochrome oxidase I, and Cytochrome b) and three nuclear genes (28S rDNA, the sodium–potassium ATPase a-subunit ‘NaK’, and Histone H3A). We employed traditional concatenated (i.e., supermatrix) phylogenetic methods, as well as three recently developed Bayesian multilocus methods aimed at inferring species trees from potentially discordant gene trees. We found strong support for two main clades within Bythograeidae: one comprising the members of the genus *Bythograea*; and the other comprising the remaining genera. Relationships within each of these two clades were partially resolved. We compare our results with an earlier hypothesis on the phylogenetic relationships among bythograeid genera based on morphology. We also discuss the biogeography of the family in the light of our results. Our species tree analyses reveal differences in how each of the three methods weighs conflicting phylogenetic signal from different gene partitions and how limits on the number of outgroup taxa may affect the results.

## Introduction

**Figure 1:**
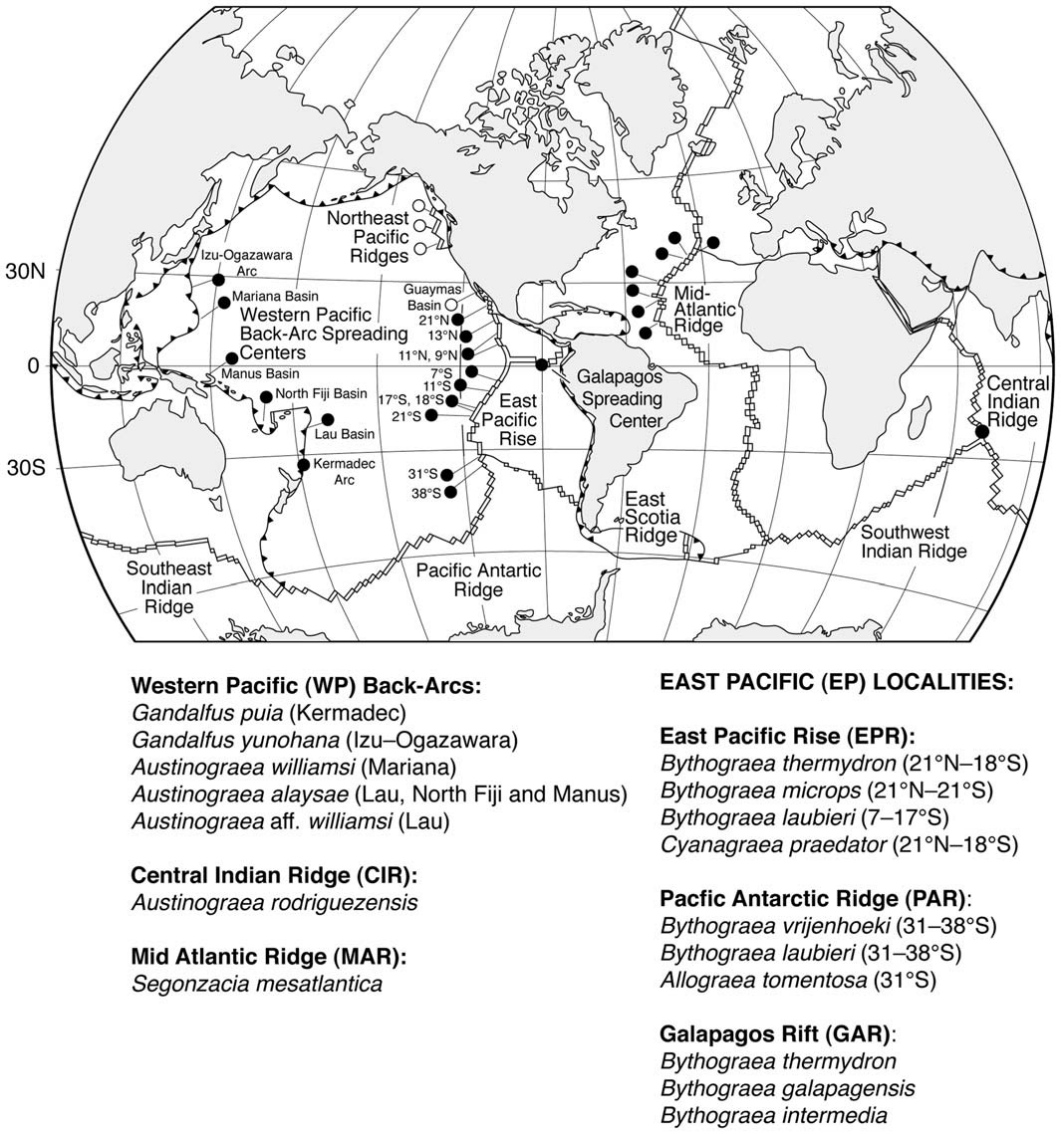
Distribution of members of the family Bythograeidae. Black circles = known vent sites with crabs. Open circles = vent sites that do not have crabs, but are referred to in text. Latitudinal range for each species indicated in parenthesis.

**Figure 2:**
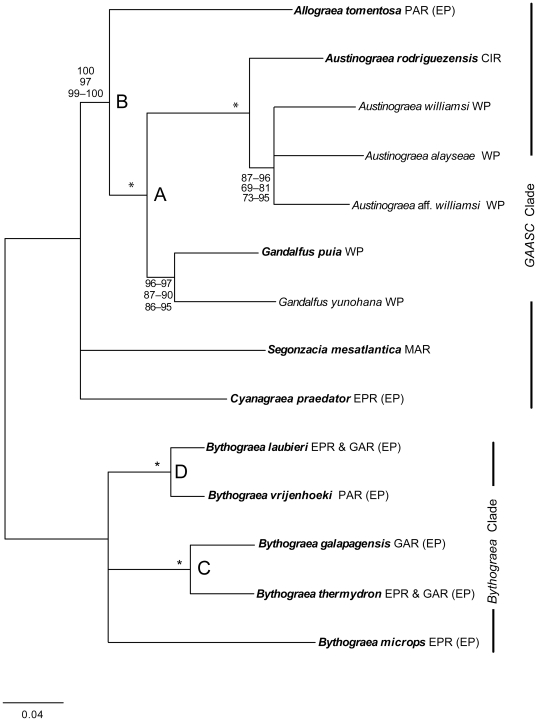
Phylogenetic relationships among members of Bythograeidae based on the concatenated analyses of six genes. Tree was rooted at the branch joining the two divergent clades (*Bythograea* and *GAASC*). Branch lengths are approximate. Bold-faced taxon labels indicate the taxa for which all six genes were obtained and included in the concatenated, species tree, and BCA analyses. The range of support values for concatenated Bayesian, GARLI, and RaxML methods (top to bottom, respectively) are depicted next to the corresponding node (support values for concatenated, species tree, and BCA analyses are shown in Table S4). Asterisks denote nodes receiving 100% support for all concatenated methods. EP = Eastern Pacific (includes: EPR = Eastern Pacific Rise; GAR = Galapagos Rift; and PAR = Pacific Antarctic Ridge). MAR = Mid-Atlantic Ridge; WP = Western Pacific; CIR = Central Indian Ridge.

Deep-sea hydrothermal vent communities contain a high proportion of endemic species, particularly at higher taxonomic levels [1]. This endemism reflects the high degree of specialization required to succeed in one of Earth's most extreme environments. The brachyuran crab family Bythograeidae Williams, 1980 (superfamily Bythograeoidea) is among the most ubiquitous and abundant group of macroinvertebrates to have colonized the deep-sea hydrothermal vents worldwide (Figure 1; [2]). It is also the only group within the diverse infraorder Brachyura (which contains 7000 valid species and subspecies in 93 families; [3]) that is endemic to this extreme environment. This is remarkable, as only one other brachyuran species from a very distant family is endemic to hydrothermal vents, but from shallow waters (i.e., *Xenograpsus testudinatus* in Xenograpsidae); and just a handful of opportunistic brachyuran species have been observed at deep-sea hydrothermal vents [2]. Understanding the evolution of this important deep-sea hydrothermal vent taxon requires knowledge on the phylogenetic relationships among its members. Although a hypothesis based on morphology has been put forth (see below), a molecular phylogenetic analysis of the Bythograeidae is lacking.

As presently diagnosed, the family Bythograeidae consists of six genera and fourteen described species (for details on the distribution of each species see Figure 1). The family is most diverse at the eastern Pacific vent systems (the East Pacific Rise, Galapagos Rift, and the Pacific-Antarctic Ridge), where it is represented by eight species belonging to three endemic genera: *Cyanagraea praedator* [4], [5]; *Allograea tomentosa* [6]–[8]; *Bythograea thermydron*; *Bythograea microps*; *Bythograea laubieri*; *Bythograea vrijenhoeki*; *Bythograea intermedia*; and *Bythograea galapagensis* (although the last two are very likely a synonymy; see [6], [9]). The Mid-Atlantic Ridge is inhabited by *Segonzacia mesatlantica* [10]. The Western Pacific back-arc basins are inhabited by *Austinograea williamsi*, *Austinograea alayseae, Gandalfus puia*, and *Gandalfus yunohana* [11]. In addition, an undescribed *Austinograea* species (*A*. affinity *williamsi*) is suspected in the Western Pacific Lau back-arc basin [2]. Finally, *Austinograea rodriguezensis* inhabits the Central Indian Ridge [12], [13]. The northeastern Pacific ridges (Explorer, Juan de Fuca, and Gorda) are the only major spreading centers that lack bythograeids. Some bythograeid species occur in large numbers at specific vent sites (e.g., *B. thermydron* and *A. williamsi*; [14], [15]), whereas others appear to be rare (e.g., *A. tomentosa* and *B. galapagensis*; [4], [6]).

In general, little is known about most bythograeid species. Most of what is known about their ecology and adaptations to hydrothermal vent environments derives from studies of *B. thermydron* (reviewed in [16]). This species is a top predator at hydrothermal vent ecosystems [17]–[19] along the East Pacific Rise and Galapagos Rift, where it is broadly distributed. It commonly occurs on aggregations (clumps) of siboglinid tubeworms and mussels, it is present at all stages of hydrothermal vent succession, and it has been observed up to 600 m away from active hydrothermal vent sites [20], [21]. This crab has evolved a number of adaptations to deep-sea hydrothermal vent environments, which include: a dependence on great hydrostatic pressures for long-term survival [22]; broad thermal tolerance ranging from ambient temperatures of 2°C to 30°C near a vent orifice [16], [22]; and physiological adaptations allowing them to cope with high sulfide and low oxygen concentrations at vents [17], [23].

Bythograeidae constitutes a taxonomically distinct group within Brachyura. Williams [24] erected this group as an independent superfamily (Bythograeoidea), because it did not fit into any of the previously recognized brachyuran families, and has been maintained as a separate superfamily since [3]. Within Bythograeidae, general morphology is extremely homogeneous, particularly the carapaces, mouthparts, thoracic sternum, walking legs, and overall facies [4], [6], [25]. According to Williams [24], Bythograeoidea exhibits some characters of Portunidae and Xanthidae, and superficial resemblance to the freshwater Potamidae. Based on comparisons of spermatozoal ultrastructure, Tudge et al. [25] and Jamieson and Tudge [26] suggest that bythograeids derive from the Xanthoidea, and that their closest relative is *Calocarcinus*, an obligate symbiont of deep-sea corals. *Calocarcinus* is currently placed within Trapezioidea, a former family of Xanthoidea [3], [27]. Based on a cladistic morphological analysis of multiple crab families, Sternberg et al. [28] suggest that Bythograeidae is sister to a clade that contains members of Potamoidea and Thoracotremata. However, a recent molecular phylogenetic study of whole mitochondrial genomes [29] found that Bythograeoidea (represented by *G. yunohana*) is closer to *Pseudocarcinus gigas* (a member of the recently established Eriphioidea, previously within Xanthoidea [3], [30]), than to members of Grapsoidea (within Thoracotremata), Portunoidea, or Potamoidea (the latter two within Heterotremata). Nonetheless, the Brachyura dataset analyzed in Yang et al.'s study was very limited, including only five superfamilies (with only one member of Xanthoidea *sensu lato*; i.e., *P. gigas*) and seven genera. Therefore, the origin of Bythograeoidea is an issue that needs further examination.

No molecular phylogenetic studies have been conducted for this group. Based on variation in eye regression and male gonopods, however, McLay [31] proposes the following phylogenetic relationships among bythograeid genera: *Allograea*+(*Segonzacia*+(*Cyanagraea*+(*Bythograea*+(*Gandalfus*+*Austinograea*)))). Herein, we examined whether this hypothesis is supported by phylogenetic analyses of mitochondrial and nuclear DNA sequences from members of the family Bythograeidae. We also discuss biogeographic and evolutionary implications of our results.

A secondary goal of this study was to compare three recently developed Bayesian methodologies designed to infer species trees based on potentially discordant gene trees. At least until recently, the most common approach for inferring phylogenies from multilocus datasets has been concatenation, which assumes that all loci share the same gene tree. However, it is well known that concatenation of loci with incongruent gene histories can lead to incorrect inference of the species tree (reviewed in [32]). To address this problem, several methods that estimate a species tree directly by incorporating heterogeneity among gene trees have been developed (reviewed in [33], [34]), but few accommodate uncertainty in gene tree estimation (reviewed in [35]). Three Bayesian methods that account for uncertainty in gene tree estimation are available: BEST (Bayesian Estimation of Species Trees; [36], [37]); *Beast (Bayesian Inference of Species Trees from Multilocus Data; [38]); and BCA (Bayesian Concordance Analysis) implemented in BUCKy (Bayesian Untangling of Concordance Knots; [39], [40]). The three methods differ in their assumptions and implementation (discussed in the Materials and Methods), and have been shown to perform differently under certain simulated scenarios [35], [38], [41]. To our knowledge, no comparisons of the results of the three methods with the same empirical dataset have been reported, but several studies have compared two of these three methods (e.g., [38], [42], [43]). This paucity is probably due in part to the difficulty of successfully implementing at least one of these methods with taxon-rich or loci-rich datasets (e.g., BEST) and the relatively recent release of *Beast. Our Bythograeidae dataset provides an empirical dataset small enough to be implemented with all three methods, and our analyses provide insight into how each method weighs both, the phylogenetic information contained in each gene and the degree of discordance among gene trees, to produce a final species tree.

## Materials and Methods

### 2.1. Biological specimens

We obtained bythograeid samples from museum collections, and other researchers and institutions (Table 1). We also included a specimen from the Lau Back-Arc Basin that has been identified as *Austinograea* affinity *williamsi* (identified by Guinot and Segonzac). These samples are the result of numerous expeditions to hydrothermal vents around the world with different underwater vehicles. The only Bythograeidae species for which we could not obtain DNA were *Gandalfus yunohana* and *Bythograea intermedia*. Nevertheless, we retrieved *G. yunohana* mitochondrial gene sequences from the whole mitochondrial genome sequence available in GenBank (Acc. No. EU647222; [29]). *Bythograea intermedia* was originally described based on six early crab stages and a megalopa, in a sample that was mixed with *B. thermydron* specimens from the original collection of the Galapagos Rift studied by Williams [24]. We tried to genetically characterize *B. intermedia* from selected specimens of this original collection, but failed to obtain adequate DNA. Adults of *B. intermedia* are not known, and it is very likely that *B. galapagensis*, which was included in the present study, is synonymous with *B. intermedia* [6], [9].

**Table 1:** Bythograeidae samples used in this study.

Species	Vent site	Lat/Long	Depth (m)	Date	Dive^a^
*Bythograea thermydron*	East Pacific Rise	11°18S; 110°32W	2791	27-XII-1998	A3323
*Bythograea microps*	East Pacific Rise	9°50N; 104°17W	2504	7-I-2006	A4207
*Bythograea laubieri*	Pacific Antarctic Ridge	31°09S; 111°55W	2338	15-I-1999	A3339
*Bythograea vrijenhoeki*	Pacific Antarctic Ridge	31°09S; 111°55W	2335	13-I-1999	A3337
*Bythograea galapagensis*	Galapagos Rift; Rose Garden	0°48N; 86°14W	2461	29-V-1990	A2224
*Cyanagraea praedator*	East Pacific Rise	17°25S; 113°12W	2582	01-I-1999	A3328
*Allograea tomentosa*	Pacific Antarctic Ridge	31°09S; 111°55W	2335	13-I-1999	A3337
*Segonzacia mesatlantica*	Mid-Atlantic Ridge	37°17N; 32°17W	1731	8-VII-1997	A3118
*Austinograea williamsi*	Mariana Back Arc Basin; Alice Springs	18°13N; 144°42E	3589	14-IX-1992	S140
*Austinograea alayseae*	Lau back-arc Basin; Valu Fa Ridge	22°13S; 176°38W	1900	22-V-1989	N BL10
*Gandalfus puia*	Kermadec Arc; Brothers Seamount	34°52S; 179°04E	1647	2-V-2005	P IV
*Gandalfus yunohana* *					
*Austinograea* aff. *williamsi*	Lau back-arc Basin; Valu Fa Ridge	22°32S, 176°43′W	1900	15-V-1989	N BL03
*Austinograea rodriguezensis*	Central Indian Ocean; Kairei	25°19S; 70°02E	2437	IV-2001	J

* Unknown collecting data for this specimen; mitochondrial sequences were obtained in GenBank Accession Number = NC_013713.

a Research Vessels: A = Alvin; N = Nautilus; S = Shinkai 6500; P IV = Pisces IV, J = Jason.

### 2.2. Molecular methods

Muscle tissue was dissected from chelae or leg segments, and DNA was extracted with the DNEasy kit (Qiagen, Inc., Valencia, CA). Published primers and PCR conditions were used to amplify three mitochondrial gene fragments and one nuclear gene fragment from: a 710-bp region of the mitochondrial Cytochrome Oxidase I gene (COI) [44]; a ∼520-bp region of the mitochondrial 16S rDNA gene (primers 16Sar/16Sbr; [45]); a 370-bp region of the mitochondrial Cytochrome b gene (Cytb; primers UCYTB144F/UCYTB270R; [46]); and a ∼677-bp region of the nuclear 28S rDNA gene (primers 28SA/28SB; [47], [48]). In addition, we successfully PCR-amplified two other nuclear genes for a subset of the species (see Results) using published primers and PCR conditions: a 382-bp fragment of the Histone H3A gene [49]; and an 870-bp fragment of the sodium–potassium ATPase a-subunit (NaK; primers NaK-F/NaK-R; [50]). PCR products were cleaned with ExoSAP (Exonuclease 1 and Shrimp Alkaline Phosphatase, USB) prior to the sequencing reaction. The BigDye® Terminator v3.1 Cycle Sequencing Kit (Applied Biosystems, Foster City, CA) was used for the sequencing reaction and samples were sequenced in an ABI PRISM® 3100 Genetic Analyzer (Applied Biosystems, Foster City, CA). We used Sequencher 4.8 (Gene Codes, Ann Arbor, MI) for editing sequences and removing primer regions.

### 2.3. Sequence Alignment

Sequences were aligned with ClustalX2.0 [51] and edited manually in MacClade 4.08 [52]. Regions of the ribosomal DNA genes for which homology assignment was questionable according to GBlocks [53], [54] with default parameters, were excluded from the phylogenetic analyses (see Table 2). In addition, a few positions adjacent to the blocks identified by GBlocks, for which we still considered homology to be questionable, were also excluded. Aligned sequences (annotated Nexus files) showing all included and excluded positions are provided in the Supporting Information.

**Table 2:** Number of included and excluded characters for the phylogenetic analyses of Bythograeidae.

Gene	No. excluded characters^a^	No. of retained characters	No. of parsimony informative characters	Best model AIC (weight)	Best model AICc (weight)	Best model BIC (weight)
28S rDNA	58	619	36	GTR+G (0.27)	GTR+G (0.26)	TIM2+G (0.39)
NaK	0	750	60	TrNef+I TrNef+G (0.13 ea)	TrNef+I TrNef+G (0.14 ea)	TrNef+I TrNef+G (0.42; 0.41)
H3A	0	324	15	HKY (0.15)	HKY (0.18)	HKY (0.41)
mitochondrial				GTR+G+I (0.64)	TIM2+G+I (0.62)	TIM2+G+I (0.99)
16S rDNA	66	472	65			
COI	0	657	168			
Cyt b	0	366	114			
Total	124	3188	458	GTR+I+G (1.00)	GTR+I+G (1.00)	GTR+I+G (0.94)

The number of parsimony-informative characters is based on included characters only. Best model selected by jModeltest according to each criterion (AIC, AICc, BIC) and its corresponding weight.

a Criteria for character exclusion are described in Materials and Methods.

### 2.4. Phylogenetic Analyses

#### 2.4.1. Outgroup identification

As explained in the Introduction, it is not clear which lineage(s) is(are) the closest relative(s) of the Bythograeidae, and thus, serve as an appropriate outgroup. Therefore, we first attempted to identify an appropriate outgroup(s) for rooting the family Bythograeidae. We initially performed phylogenetic analyses on four datasets that included our samples of Bythograeidae and *Calocarcinus africanus* (which has been suggested to be the closest relative of bythograeids [25] and [26]), as well as numerous members of Brachyura for which DNA sequences are reported in GenBank. These datasets were: (1) concatenated 16S rDNA+COI+Cytb mitochondrial genes [29]; (2) nuclear H3A; (3) concatenated nuclear H3A+mitochondrial 16S rDNA [55]; (4) nuclear 28S rDNA; and, (5) nuclear NaK [50]. Phylogenetic methods and model selection are described below and in Supporting Table S1 (aligned datasets are deposited in the Supporting Information Datasets S1, S2, S3, S4, S5). Species tree analyses were not attempted for these datasets because of the small number of shared genes across taxa.

#### 2.4.2 Ingroup analyses: family Bythograeidae

The outgroup identification analyses did not allow identification of the closest relative(s) of bythograeids (see Results). However, these analyses supported the existence of two separate and divergent clades within the Bythograeidae: the genus *Bythograea* vs. a group formed by the other five bythograeid genera (hereafter *GAASC* clade; see Results). The outgroup identification analyses also failed to recover the monophyly of Bythograeidae, but alternative relationships were not supported either (see Results). Thus, given that morphological evidence strongly supports the monophyly of the family (i.e., extremely homogeneous within, and very distinct from other brachyurans; [2], [24], [25]), and that our phylogenetic analyses do not support or refute it, we used each of these two clades within Bythograeidae to root the other. Taxa outside Bythograeidae were not included because they are likely more distant and, thus, could increase the probability of long-branch attraction.

Phylogenetic analyses of members of the Bythograeidae were conducted on datasets of the combined genes. Because not all genes were obtained for all taxa, some analyses were performed on subsets of taxa. Nevertheless, we obtained all genes for at least one species per genus in the *GAASC* clade, and for all five *Bythograea* species (i.e., the 10-taxon dataset; see below). To determine the most appropriate model of DNA substitution we used jModeltest v0.1.1 [56] to evaluate 88 substitution models with full likelihood optimization, under the Akaike Information Criterion (AIC), corrected AIC(c), and Bayesian Information Criterion (BIC) (selected models and corresponding weights are shown in Table 2). We used these models or the closest more complex model available to conduct maximum-likelihood (ML) searches and Bayesian analyses (see Table S2). However, when a proportion of invariable sites (I) and a Gamma distribution of rates among sites (G) was selected according to jModeltest, we excluded parameter I because of the potential problems with estimating G+I simultaneously (see RaxML manual and pages 113–14 in [57]).

For the ML analyses, we used two different programs: (a) RaxML 7.2.6 [58]–[60]; and (b) GARLI v.0.96beta8 [61], as implemented in a computer cluster (brazos.tamu.edu). In RaxML, we used three different partitioning schemes: a single partition; partition by gene; and partition by linkage groups (i.e., mitochondrial genes in a single partition, and each nuclear gene in its own partition). For the Bayesian analyses, we used two approaches. The first was the traditional concatenation method (a.k.a., supermatrix), which does not accommodate variation in coalescent histories among unlinked loci: (a) MrBayes parallel version 3.1.2 [62], [63] with a single data partition; and (b) BayesPhylogenies parallel version 2.0.2 [64]. BayesPhylogenies allows one to assume different numbers of data partitions (“patterns”), but without a priori assignment of sites to a partition. We tested 1–6 partitions and identified the best partitioning scheme according to Bayes Factors following Kass and Raftery [65]. Marginal posterior probabilities were estimated in Tracer v.1.5 [66], following Newton and Raftery [67] modified by Suchard et al. [68].

The second Bayesian analysis approach was with three methods that attempt to infer a species tree based on potentially discordant gene trees: BEST v.2.3.1 [36], [37]; *Beast v1.6.1 [38]; and BUCKy v.1.4.0 [39], [40]. BEST and *Beast are species tree methods, which estimate species tree topology, divergence times, and population sizes from gene trees under a multispecies coalescent model. They both assume that differences in gene trees are due to incomplete lineage sorting, and estimate gene and species trees jointly. The main differences between these two coalescence-based methods are: (a) BEST assumes a constant population size along each branch, whereas *BEAST implements several population size models (default = piecewise linear and constant root); (b) BEST requires an outgroup (only one outgroup taxon is allowed), whereas *BEAST allows more than one outgroup, but does not require any; and (c) BEST assumes a species tree uniform prior, whereas *Beast assumes a Yule (default) or birth-death model. Because of the outgroup restriction in BEST, we arbitrarily selected *Bythograea thermydron* as the outgroup for the *GAASC* clade; and *Gandalfus puia* as the outgroup for the genus *Bythograea* in two separate analyses; each with six taxa (i.e., five ingroup and one outgroup). In addition, to evaluate whether discrepancies in BEST and *Beast were the result of different outgroup sampling, we also conducted the *Beast analyses with the same six taxa used in BEST (see Results). The Bayesian Concordance Analysis (BCA), implemented in BUCKy [39], [40] makes no assumption regarding the reason for discordance among gene trees (e.g., incomplete lineage sorting, recombination, horizontal gene transfer). It is not a species tree method because it does not assume a multispecies coalescent. Instead, it uses a non-parametric clustering of genes with compatible trees, and reconstructs the primary concordance tree from clades supported by the largest proportions of genes (accounting for uncertainty in gene tree estimates; which are estimated in MrBayes). Although the primary concordance tree is not necessarily the species tree (e.g., in the “anomaly zone”; see [33]), it is expected to be similar or isomorphic to the species tree under many circumstances. Hereafter, for simplicity, we also refer to BUCKy as a species tree method.

Clade support was determined based on non-parametric bootstrap support (BP) for ML analyses (at least 1000 replicates), on Bayesian posterior probabilities (PP) for Bayesian analyses, and on concordance factors (CF) for BUCKy, which represent the proportion of genes that truly have the corresponding clade in their trees. Fifty-percent majority rule consensus trees were summarized with the Sumtrees command implemented in DendroPy-3.7.1 [69]. For the Bayesian analyses, the number of Markov Chain Monte Carlo (MCMC) generations, the sampling frequency, and the number of independent runs for each analysis are shown in Table S2. All other parameters not shown were default or the ones specified in the *Beast tutorial (http://beast.bio.ed.ac.uk/Tutorials; Oct 8, 2010). For BUCKy, we tested several reasonable priors for the discordance parameter (α = 0.01, 0.5, 1, 2, 10, and 1000), given the number of genes, number of taxa [70], and the observation that at least three relationships were highly concordant among genes (see Results).

To determine whether the MCMC had reached convergence on a stationary distribution and whether a sufficient sample of the stationary distribution had been obtained, we used the following criteria: (a) Stable posterior probability values (all methods except BUCKy); (b) a high correlation between the split frequencies of independent runs (all methods except BUCKy) as implemented in AWTY [71]; (c) small and stable average standard deviation of the split frequencies of independent runs (MrBayes and BEST only); (d) Potential Scale Reduction Factor close to 1 (MrBayes); and (e) an Effective Sample Size (ESS)>200 for the posterior probabilities (all methods except BUCKy, as evaluated in Tracer v. 1.5; [66]). Samples prior to reaching a stationary posterior distribution were discarded (i.e., “burnin”).

## Results

### 3.1. Alignment and Datasets

We obtained the sequences for all six genes from most taxa with the following exceptions: for *Austinograea williamsi*, *A. alayseae* and *A.* aff. *williamsi*, we could not obtain the COI, H3A, and NaK genes; and, for *Gandalfus yunohana*, only the mitochondrial genes were available, leaving all five *Bythograea* spp. and one representative per genus for the remaining five genera with all six genes sequenced ( = 10 taxa). All new sequences have been deposited in GenBank under Acc. Nos. JQ407410-JQ407489, and our alignments have been deposited as Nexus files in Supporting Information Datasets S6, S7, S8.

### 3.2. Outgroup identification

Our results based on three single-gene datasets—i.e., 28S rDNA, Nak, H3A—, and two concatenated datasets—i.e., 16S rDNA+COI+Cytb (mitochondrial; mt), and 16S rDNA+H3A—, suggested that the genus *Bythograea* is monophyletic (Supporting Table S3), with bootstrap and posterior probability values between 99–100% for all datasets except H3A, for which support was lower (i.e., 70–74% ML bootstrap support; Bayesian analyses were not conducted for this dataset due to the large number of taxa; n = 284; Supporting Table S1). The remaining genera of the Bythograeidae (*Gandalfus, Austinograea, Allograea, Segonzacia, Cyanagraea*) formed a monophyletic clade (the *GAASC* clade) with 88–100% support in the 16S rDNA+COI+Cytb dataset; 79–100% for the Nak dataset; and 61–90% support in the 16S rDNA+H3A dataset. Bootstrap support for the *GAASC* clade in the H3A dataset ranged from <50% to 65% in the ML analyses (Supporting Table S3). The analyses of 28S rDNA failed to recover the *GAASC* monophyly with ≥50% bootstrap or posterior probability. Alternative relationships to the *GAASC* monophyly were not supported with either the H3A or the 28S rDNA dataset. Relationships among the genus *Bythograea*, the *GAASC* clade, and the other Brachyuran lineages examined, were not well resolved and effectively resulted in a polytomy (Supporting Figure S1), suggesting that none of these datasets contain sufficient phylogenetic signal to examine the monophyly of the Bythograeidae and identify its closest relatives. Consequently, to infer relationships within the two clades of Bythograeidae, no outgroup outside Bythograeidae was included in the subsequent phylogenetic analyses. Instead, the resulting trees were rooted at the branch that separates the two clades: *Bythograea* and the *GAASC*.

### 3.3. Phylogenetic analyses of the Bythograeidae-only dataset

After exclusion of 58 positions from the 28S rDNA gene and 66 positions from the 16S rDNA gene due to uncertainty in the alignment and/or missing data, the Bythograeidae six-gene dataset contained 3188 characters of which 458 were parsimony-informative (Table 2). We initially conducted concatenated and species tree phylogenetic analyses of the Bythograeidae on a dataset including all four linkage groups (i.e., six genes: the nuclear 28S rDNA, H3A, and NaK; and the mitochondrial 16S rDNA, COI and Cytb genes). Discrepancies among linkage groups were revealed by the analyses of individual linkage groups (only MrBayes results are reported, but similar results were obtained with Garli and RaxML), and in the species tree analyses (described in detail below). Thus, to examine the effect of linkage group on the coalescence species tree analyses (BEST and *Beast), we also conducted these analyses on datasets of all four possible combinations of three linkage groups (i.e., excluding one linkage group at a time; see Supporting Table S4).

#### 3.3.1. Model selection

For the concatenated datasets, the best substitution model according to the three selection criteria (AIC, AICc and BIC) was the General Time Reversible plus gamma plus a proportion of invariable sites (GTR+G+I; Table 2). The best models selected for each separate linkage group ranged from relatively simple (e.g., HKY for H3A; Table 2) to complex (e.g., GTR+G+I for the mitochondrial genes). Therefore, for the phylogenetic analyses, we used the best model(s) according to all three criteria, or the closest model if the best model was unavailable in a program (see Table S2). Furthermore, as explained in the Materials and Methods, when the best model included G+I, I was not included.

#### 3.3.2. Phylogenetic Relationships within Bythograeidae

Figure 2 depicts the relationships supported by >50% bootstrap and posterior probability values in all the concatenated supermatrix analyses. All other nodes have been collapsed. Clade support results of the supermatrix and species tree analyses are summarized in Supporting Table S4. All our phylogenetic analyses of the family Bythograeidae recovered a split with 100% support (not shown) between the genus *Bythograea* and the remaining five bythograeid genera (*GAASC* clade), which is in agreement with our “Outgroup Identification” results. The ranges of Kimura-2-Parameter divergences for the nuclear genes combined and for mitochondrial (mt) genes combined, respectively were: (a) within *Bythograea* = 0.12–2.41% and 4.36–13.88%; (b) within the *GAASC* clade = 1.5–3.23% and 13.26–16.21%; and (c) between *Bythograea* and the *GAASC* clade = 5.36–6.97% and 15.89–20.15% (Supporting Table S5).

#### 3.3.2.1. Clade GAASC

The monophyly of the genus *Austinograea* received 100% support in all concatenated analyses of the 16S rDNA, COI, Cytb, and 28S rDNA (Fig. 2); the other genes were not obtained for *A. williamsi*, *A. alayseae* and *A.* aff. *williamsi*. *Austinograea* was divided into two lineages: *A. rodriguezensis* from the Central Indian Ocean and the remaining *Austinograea* species from the Western Pacific. The relationships among the Western Pacific *Austinograea* were not resolved. The two species of *Gandalfus* formed a monophyletic group with 86–97% support; this relationship was based on the three mt genes only.

All of our analyses recovered the monophyly of *Gandalfus* and *Austinograea* (*G-Au*; clade A) with high bootstrap proportions (BP), Bayesian posterior probability (PP) and concordance factor (CF) (Fig. 2; Table S4). Most of our analyses recovered the monophyly of *Gandalfus-Austinograea-Allograea* (*G-Au-Al*; clade B). This relationship was highly supported (96%) by the mitochondrial genes alone (Table S4F) and by the concatenated analyses (≥97%; Table S4A). Most species tree analyses recovered this relationship, but support was weaker. The only analyses that failed to recover this relationship were those of the individual nuclear genes (Table S4F), the BEST analyses that included all six genes (four linkage groups; Table S4A), and the BEST and *Beast analyses that excluded the mitochondrial (mt) genes (i.e., one of the three-linkage-group analyses; Table S4E). In neither case did alternative relationships receive clade support >75%. The concordance factor (CF) for the *G-Au-Al* clade was high (≥94% for all á values tested; Table S4G). The number of taxa in the *Beast analyses influenced the degree of support for this relationship, with higher support when all 10 taxa were included compared to 6 taxa only (78% vs. 68%, respectively; Table S4A). Additionally, removing NaK increased the support for the *G-Au-Al* clade to 85% and 80%; respectively (Table S4B). However, removal of any of the other nuclear genes had little effect in *Beast (Table S4C and D). In BEST, the number of linkage groups influenced the degree of support for this clade. Removal of any one of the nuclear genes (i.e., leaving the mt linkage group and two of the three nuclear genes; Table S4B, C, and D), recovered the *G-Au-Al* clade, albeit with variable support depending on the excluded nuclear gene (78–99% PP). Support for this clade was 99% when NaK was removed; 98% when 28S rDNA was removed; and 78% when H3A was removed. Removal of the mt partition resulted in <50% support for the *G-Au-Al* clade for both, BEST and *Beast, but alternative relationships were not recovered at ≥50% PP (Table S4E).

Relationships of the *G-Au-Al* clade to the remaining members of the *GAASC* clade (i.e., *Segonzacia* and *Cyanagraea*) were less straightforward. Monophyly of *Segonzacia* and *Cyanagraea* (hereafter *S-C*) was recovered with low-to-moderate support in the individual linkage group analyses of 28S rDNA and the mt linkage group (70 and 67% support; respectively), and in few of the concatenated analyses (Table S4A). However, concatenated analyses of the mitochondrial+28S rDNA partitions (not shown in Table S4), including all 14 Bythograeidae species examined in this study, obtained moderate-to-high support for the *S-C* monophyly: 96–97% for Bayesian analyses; 79–81% for RaxML; and 69% for Garli. Similarly, all *Beast analyses that included the mitochondrial linkage group recovered the *S-C* monophyly with 80–94% support (see Table S4A–D). Among these analyses, support for the *S-C* was higher for the 10-taxa dataset than for the 6-taxa dataset (90 and 82%; respectively; four-linkage groups; Table S4A), and for the three-linkage group dataset that lacked the NaK partition (92–94%; Table S4B). BEST analyses, however, did not recover this relationship at all. In two cases, BEST recovered the relationship *G-Au-Al*+*Segonzacia* (*G-Au-Al*-*S*) with weak support (63 and 52%; Table S4B and D; respectively); and in one case it recovered the relationship *G-Au-Al*+*Cyanagraea* (*G-Au-Al*-*C*) also with weak support (60%; Table S4E). BUCKy obtained a CF of 71–76% for the relationship *G-Au-Al*+*Cyanagraea* (*G-Au-Al*-*C*), whereas a CF of 21–25% for the *S-C* monophyly (Table S4G). These results suggest a high degree of discordance among our datasets and phylogenetic methods. Therefore, our analyses fail to resolve with confidence the relationships among *G-Au-Al*, *Cyanagraea*, and *Segonzacia*.

#### 3.3.2 Genus *Bythograea*

Within the genus *Bythograea*, all analyses consistently recovered two pairs of sister species, each with high support (Table S4; Fig. 2): *B. vrijenhoeki–B. laubieri* (clade D); and *B. thermydron–B. galapagensis* (clade E). The placement of *B. microps*, however, was not resolved. Only the *Beast analyses provided moderate-to-high support for the monophyly of *B. vrijenhoeki–B. laubieri–B. thermydron–B. galapagensis* (*T-G-V-L*; 88–92% and 69%; in the presence and absence of NaK; respectively). BEST either failed to resolve this relationship or recovered the alternative relationship *B. vrijenhoeki–B. laubieri–B. microps* (*V-L-M*), with low to moderate support depending on the excluded partition (51–83%). Despite conducting multiple runs with the longest MCMC chains permitted by *Beast (i.e., 10^9^), the analyses of the genus *Bythograea* with *Gandalfus* as outgroup (i.e., 6-taxa *Bythograea* in Table S4) did not seem to reach a stationary posterior distribution because posterior probabilities appeared to continue increasing, and thus, were not reported. BUCKy estimated a CF of 50–51% for the *V-L-M* clade and of 47–48% for the *T-G-V-L* clade, clearly illustrating the strong discordance among linkage groups for this relationship. Therefore, our data failed to resolve the position of *B. microps* relative to the two *Bythograea* species pairs.

## Discussion

### 4.1. Comparison of Bayesian Species Tree Methods

The development of methods that can incorporate discordant coalescent histories among unlinked loci into the inference of a common species tree is of great interest. Of the methods available, Bayesian approaches are particularly appealing because they can readily incorporate uncertainty in the gene trees, whereas ML-based methods (e.g., STEM; [72]) are unable to do so in a computationally feasible manner. Three Bayesian methods have become available relatively recently: BEST [36], [37]; *Beast [38]; and BUCKy [39], [40]. Studies comparing the three methods based on simulated data suggest differences in performance [35], [38], [41]. Comparisons of the three methods with empirical data are lacking, although a few comparisons of two out of the three methods suggest differences (e.g., [38], [42], [43], [73], [74]; discussed below). Our Bythograeidae dataset, which is small enough to be implemented with all three methods, and contains gene tree incongruence, allowed us to gain insight into how each method incorporates both, the phylogenetic information contained in each gene, and the degree of discordance among gene trees.

In our study, BEST, *Beast, and BUCKy behaved quite differently depending on the particular clade. For example, when the mitochondrial (mt) genes were included, *Beast recovered the *G-Au-Al* clade, although support was variable depending on taxon sampling and linkage group combination (e.g., 78% for all genes-10 taxa; 68% all genes-6 taxa; and 85% all genes except NaK-10 taxa). BEST only recovered this relationship if the mt linkage group was combined with only two (out of the three) nuclear genes, regardless of which ones (78–99% support). Since the *G-Au-Al* clade was strongly supported by the concatenated analyses, the CF, and the mt linkage group alone, it appears that *Beast gives more weight to the signal from the linkage group with the largest number of informative sites (i.e., the mt partition) than BEST, which appears to dilute the signal from the mt dataset. The BCA analysis (BUCKy), which uses the Bayesian posterior sample of individual gene trees obtained with MrBayes, revealed a CF of 94–100% for the *G-Au-Al* clade, suggesting that the conflicting signal of the nuclear genes is not strong enough to counter the high individual support (i.e., 96%) of the mt partition for this relationship.

Differences among the three species tree methods were also observed in the relationships among *S*, *C*, and the *G-Au-Al* clade, where discordance between gene trees was evident in the analyses of individual partitions. Two of them (28S rDNA and mt) moderately supported the *S-C* monophyly (70% and 67% PP; respectively), whereas the other two (H3A and NaK) moderately supported the alternative *G-Au-Al-C* monophyly (68% and 63% PP; respectively). BUCKy detected substantial discordance among gene trees, with the *G-Au-Al-C* monophyly supported by approximately the equivalent to three out of four partitions (CF = 71–76%), whereas the *S-C* monophyly by approximately one out of four partitions (CF = 21%). Both, BEST and *Beast, recovered *G-Au-Al-C* (60 and 62% PP; respectively) only when the mt partition was removed (i.e., the three nuclear genes only). In contrast, *Beast recovered the *S-C* monophyly with 80–94% support in all analyses that included the mt partition. These results highlight that different conclusions might be reached depending on the method of choice; e.g., had we only conducted *Beast analyses of the four linkage-group dataset, we may have concluded with relatively high confidence that the most likely relationship was *S-C*; a relationship that is not favored by the BEST or BUCKy analyses of the same dataset.

Inferences about relationships within the genus *Bythograea* varied between BEST and *Beast also; they only agreed in one instance (i.e., in the analyses excluding the mt partition, both recovered the *T-G-V-L* clade; Table S4E). The discordant relationships were supported by >80% PP for each method in one of the datasets (Table S4C). The influence of taxon sampling on these discrepancies could not be evaluated (i.e., due to lack of convergence of the 6-taxon *Beast analyses). In this regard, it would be useful for *Beast to allow longer runs (>10^9^ generations). The CFs for the two discordant relationships within the genus *Bythograea* (*V-L-M* and *T-G-V-L*) were relatively low and similar (51% and 44%; respectively), suggesting a strong discordance of the NaK vs. the mt and 28S rDNA partitions.

The discordant results observed in our study between *Beast and BEST were unexpected, because the two methods appear to be very similar (i.e., both are Bayesian methods that assume a multispecies coalescent). To our knowledge, only one empirical dataset has been used to compare the results of BEST and *Beast. Belfiore et al. [75] used BEST to examine the relationships among eight species of the rodent genus *Thomomys*, based on seven loci. A subset of this dataset (i.e., seven species) was then subjected to *Beast analyses by Heled and Drummond [38]. Unfortunately, few conclusions can be drawn from the comparison of these two studies due to the limited resolution at several nodes and differences in the number of taxa examined. An important observation, however, is that *Beast, which does not require an outgroup for rooting purposes, was unable to place the root at the branch joining the ingroup (*Thomomys*) and outgroup (*Orthogeomys*), unless the monophyly of the ingroup was specified a priori [38].

Although our dataset lacks the multiple alleles per species recommended for coalescent-based species tree estimations, it illustrates differences between the approaches implemented in BEST and *Beast that will likely be relevant, even with the inclusion of multiple alleles per species. First, outgroup taxon sampling influenced the results of *Beast. In every instance evaluated, posterior probabilities for *G-Au-Al* in *Beast with one outgroup (*B. thermydron*; 6-taxon dataset) were lower than with five outgroup taxa (five *Bythograea* species; 10-taxon dataset). A possible explanation is that the branch that joins the *GAASC* clade and *B. thermydron* is subject to “long-branch attraction”, and that addition of other divergent members of the genus *Bythograea* effectively breaks this long branch, thereby reducing the effect of long-branch attraction (see [76]). Although we did not examine the effect of using different members of the genus *Bythograea* as the single outgroup because it was not computationally feasible, it is likely that similar results would have been obtained, as their divergences (i.e., genetic distances) from the *GAASC* clade are quite similar (Supporting Table S5). If our observations of increased *G-Au-Al* clade support in *Beast with more outgroup taxa are due to a reduction of long-branch attraction, it is conceivable that long-branch attraction could have affected our BEST estimates. In this regard, the ability to use more than one outgroup in BEST would constitute a significant improvement to this method.

Second, signal from the different linkage groups appears to be weighted differently by *Beast and BEST. In general, *Beast seems to assign more weight to: (a) partitions that have more informative characters (e.g., the mt partition), even when the discordant relationships are not highly supported by any partition (e.g., relationships among *S*, *C*, and *G-Au-Al*); and/or (b) partitions that have high individual support for a relationships (e.g., the mt partition for the *G-Au-Al* clade), when other partitions provide relatively low support for discordant relationships. In contrast, BEST appears to weigh partitions more evenly, by diluting the signal of a “strong” partition if multiple partitions support, albeit weakly, alternative relationships. The difference in these behaviors may be due to differences in priors (e.g., constant population size vs. piecewise linear and constant root; species tree uniform prior vs. Yule or birth-death model). In our dataset, the mitochondrial partition contains more informative sites than any of the individual or the combined nuclear partitions, a common situation in many datasets [77]. Dilution of the signal from a “strong” partition would be problematic if the “strong” partition more closely reflects the species tree than multiple “weak” partitions.

Other studies have compared results from BUCKy and BEST and reported some disagreement between them. Cranston et al. [74] examined a dataset of 162 genes in six species of rice with concatenated analyses and BUCKy, and subsets of 10, 20, 30, 40 genes with BEST (analyses of the full 162-gene dataset failed to converge). Although several of the BEST results agreed with the results from BUCKy and the concatenated analyses, several recovered different topologies. In general, they found that the results of BUCKy were more similar to those of the concatenated analyses than to the results of BEST. In our study, BUCKy obtained a high CF for a node supported by *Beast, whereas BEST failed to resolve it (i.e., *G-Au-Al* clade; all four linkage groups). The remaining clades with high gene tree discordance (i.e., <76% CF) were essentially unresolved by BEST. Finally, Lee et al. [73] compared the results of concatenated, BEST, and BUCKy methods, as well as two non-Bayesian species tree methods — STAR [78] and MDC [79]–[81]— for a dataset comprising 18 loci in 25 species in the bird family Maluridae. Despite major discordances among gene trees, the results of BEST, BUCKy, and STAR were generally similar to each other and to the concatenated tree, except for two major discrepancies between BEST and BUCKy/STAR. Incongruent results among species tree methods in our study and in the few studies that have used empirical data to compare these methods, caution against the use of a single species tree method. Further examination of these methods with both, empirical and simulated data, is needed to better understand the nature of the differences among these methods.

### 4.2. Outgroup identification

Although we conducted analyses to identify one or more appropriate outgroups for Bythograeoidea, identification of the closest relative to Bythograeidae within Brachyura was out of the scope of this study. This is a major task that will only be accomplished with extensive representation of many brachyuran genera, families, and superfamilies. Unfortunately, our analyses provide no clues on this issue, showing, in general, low resolution with many taxa converging to basal polytomies. This is an indication that many informative characters are needed to establish relationships in higher taxonomic levels of Brachyura. For example, our analyses of the three mitochondrial genes (Supporting Figure S1B), which included most of the brachyuran taxa used by Yang et al. [29], consistently recovered the two main Bythograeidae clades, but lacked deeper resolution. However, based on complete mitochondrial genomes, Yang et al. [29] obtained a well-resolved tree, in which *G. yunohana* (Bythograeoidea) is closer to *Pseudocarcinus gigas* (Eriphioidea, previously within Xanthoidea [3]), than to members of other families previously suggested to be close to Bythograeoidea (i.e., Portunoidea, Potamoidea, and Grapsoidea). Thus, it appears that many more informative sites are needed to find the level of resolution achieved by Yang et al. [29].

A close affinity between Bythograeoidea and Xanthoidea *sensu lato* (i.e., including also taxa that were previously within Xanthoidea, but have been recently moved to newly erected superfamilies; see below) has been suggested [2], [24], [27], [29]. Xanthoidea *s. l.* is a speciose taxonomic group that comprises many families and genera, and has been subjected to major taxonomic revisions [3], [27]. For example, genera previously assigned to Xanthoidea, are now assigned to the recently erected superfamilies Eriphioidea, Trapezioidea, Carpiliioidea, and Pilumnoidea. In addition, the family Pseudorhombilidae, long associated with the goneplacids, has been moved to Xanthoidea, which also includes the families Panopeidae and Xanthidae (the latter is one of the largest families in Brachyura with 13 subfamilies, 124 genera and 639 species). Our analyses of 16S rDNA+H3A (Supporting Figure S1C) included members of Xanthoidea, Trapezoidea, Eriphioidea, and Pilumnoidea, but the relationships among them and with Bythograeoidea were largely inconclusive, resulting in basal polytomies. In these analyses, *Calocarcinus*, suggested to be the closest relative of Bythograeoidea [25], was placed in a clade that includes *Philippicarcinus*, which is sister to a clade that includes *Trapezia* and *Quadrella*. This is consistent with the present taxonomy [3] that includes *Calocarcinus* and *Philippicarcinus* in the subfamily Calocarcininae, and these genera along with *Trapezia* and *Quadrella* in the family Trapeziidae (within Trapezioidea). All our “outgroup identification” analyses included *Calocarcinus africanus* and we found no indication of this species being the closest relative to Bythograeidae.

We found strong support for two monophyletic groups within Bythograeidae: genus *Bythograea* vs. the five remaining genera *GAASC*. Nevertheless, monophyly of the family (i.e., *Bythograea*+*GAASC*) was not recovered in the analyses of nuclear and mitochondrial genes that included other Brachyuran crabs. Although this could indicate two independent colonizations of deep-sea hydrothermal vents, we consider this interpretation unlikely. First, bythograeids are extremely homogeneous in general morphology, and constitute a distinct and robust taxonomic group within Brachyura [4], [6], [25]. Second, colonization of hydrothermal vents has been extremely rare among brachyuran crabs [2], limited to bythograeids in the deep-sea hydrothermal vents and to *X. testudinatus* in shallow water hydrothermal vents. Thus, two independent radiations into deep-sea hydrothermal vents and subsequent morphological convergence between these two lineages appear improbable. Our failure to recover the monophyly of the family may just reflect a general lack of resolution at this and deeper phylogenetic levels for the markers used, as illustrated by the basal polytomy recovered for relationships among other brachyuran taxa.

The unequivocal division between *Bythograea* and the *GAASC* clade facilitated our phylogenetic analyses, by allowing us to root each clade with the other. The division between these two clades was supported by the “outgroup identification” analyses; and by the 100% support received by the branch that splits the two groups, in all the Bythograeidae-only analyses.

### 4.2 Phylogenetic Relationships within Bythograeidae

**Figure 3:**
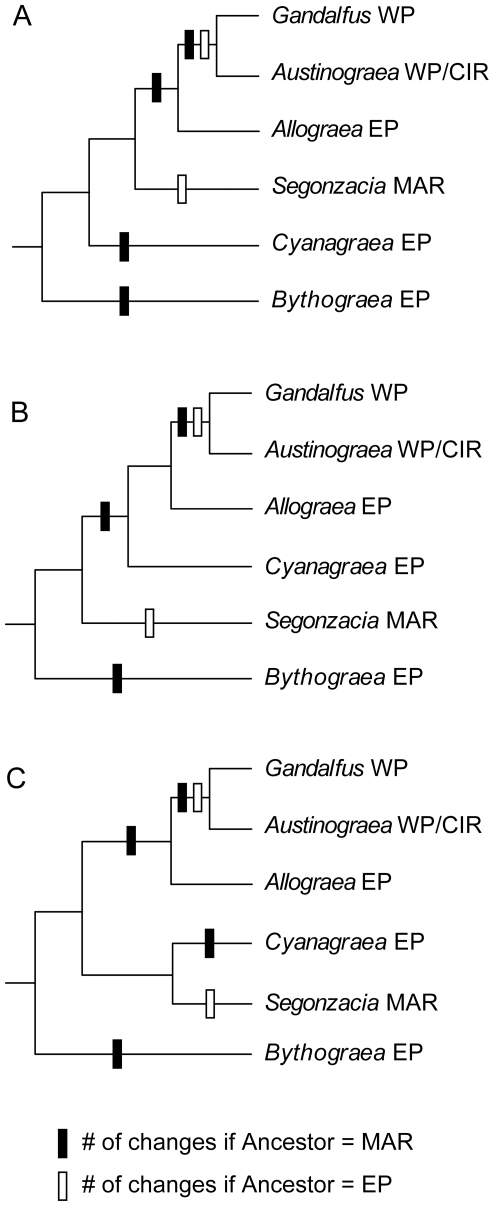
Possible biogeographic scenarios for the three alternative relationships among bythograeid genera. These scenarios take into account the phylogenetic uncertainty in the *GAASC* clade for the relationships among *Cyanagraea*, *Segonzacia*, and (*Allograea*+*Gandalfus*+*Austinograea*). Bars on nodes depict inferred geographic region shifts if ancestor of Bythograeidae was in the Eastern Pacific (EP; white bars) or in the Mid-Atlantic Ridge (MAR; black bars). Alternative equally parsimonious reconstructions exist in some cases, but are not shown for simplicity. Inferred shifts if ancestor was in Western Pacific (WP) or Central Indian Ridge (CIR) are not depicted, but they would require the largest number of shifts in any of the three topologies.

Our results do not completely solve the phylogenetic relationships among members of Bythograeidae. However, they provide important clues that advance our understanding on the evolution and biogeography of this group. First, the deep basal split between *Bythograea* and the other genera. Second, the monophyly of *Austinograea*-*Gandalfus*. Third, the monophyly of *Allograea*-*Austinograea*-*Gandalfus*, which we regard as a highly likely relationship considering that most analyses recovered it, and that none of the multilocus analyses (concatenated or species tree) supported an alternative relationship. Only the NaK and 28S rDNA individual datasets recovered alternative relationships with >50% support: NaK analyses *G-Au-C* with 75% PP and 28S rDNA analyses recovered *G-Au-S-C* with 72% PP. NaK, however, may be a problematic marker, as it is reported to have multiple copies in many taxa, including invertebrates [82], [83], and we are uncertain whether this is the case in our study. Fourth, the presence of two pairs of sister species within *Bythograea*: (*B. thermydron*-*B. galapagensis*) and (*B. laubieri*-*B. vrijenhoeki*). This is consistent with a previous taxonomic study [6] that also recognized these pairs of sister species based on morphological characters. Fifth, the early divergence between *A. rodriguezensis* (Central Indian Ocean) and Western Pacific *Austinograea* lineages. Although the relationships between *G-Au-Al* and the other two genera *Segonzacia* and *Cyanagraea* still need to be solved, our analyses reduced to three the number of possible topologies for the relationships among Bythograeidae genera (Figure 3). Concatenated analyses of mitochondrial and 28S rDNA partitions, however, recover the *S-C* monophyly with high support. The relationships between *B. microps* and the two clades of *Bythograea* sister species, and the relationships among Western Pacific *Austinograea* species, also need to be resolved.

### 4.3 Undescribed *Austinograea* species

Genetic divergence between the Lau Back-Arc Basin specimens identified as *Austinograea* aff. *williamsi* and the other West Pacific *Austinograea* suggests that this specimen warrants recognition as a new species, as suggested by Guinot and Segonzac [2]. Uncorrected nucleotide divergence for the mitochondrial 16S rDNA gene between *A.* aff. *williamsi* and *A. williamsi* is 5.5%; between *A.* aff. *williamsi* and *A. alayseae* is 7%; whereas between *A. williamsi* and *A. alayseae* is 5.9%.

### 4.4 Comparison with phylogenetic hypothesis based on morphology

Our results provide mixed support for McLay's [31] phylogenetic hypothesis for the Bythograeidae based on morphological characters: (*Allograea*+(*Segonzacia*+(*Cyanagraea*+(*Bythograea*+(*Gandalfus*+*Austinograea*))))). In agreement with McLay, we found a sister relationship between *Gandalfus* and *Austinograea*. However, we find evidence against *Allograea* being sister to all the other genera (including *Bythograea*), and against *Bythograea* being sister to (*Gandalfus*+*Austinograea*). The morphological traits used by McLay [31] provide limited phylogenetic information to solve the relationships among Bythograeidae genera. These include the relative lengths of the first (G1) and second (G2) gonopods, whether G1 has setae or spines, and degrees of eye regression (Table 3). According to these gonopod characteristics only two groups can be defined: (1) *Segonzacia*+*Cyanagraea*+*Bythograea*, which have G2≥G1, spines absent on G1, and setae generally present on G1; and, (2) *Austinograea*+*Gandalfus*, which have G2≤G1 and spines present and setae absent on G1. No information on the gonopods is available for *Allograea* since males of this genus have not been collected. McLay hypothesizes that G2>G1 is the ancestral condition in bythograeids, which is consistent with our results. As for the eye regression, only two groups can be recognized: (1) *Segonzacia*+*Cyanagraea*+*Allograea*+*Bythograea*, which have mobile eyes with cornea; and (2) *Austinograea*+*Gandalfus*, which have fixed, recessed eyestalks with cornea vestigial, unpigmented, or absent. Therefore, the only reliable phylogenetic inference that can be drawn from these traits used by McLay is the monophyly of *Austinograea*+*Gandalfus*, which is confirmed by our results. This monophyly is confirmed by another morphological synapomorphy: the thoracic sternum/pterygostome junction present in *Austinograea* (figs. 6a, 8b in [84]) and in *Gandalfus* (Guinot, unpublished data), but absent in the other bythograeids (figs. 1b, 6b, c, 8a, c, 15b in [84]); with a varying degree of junction in *Austinograea* and *Gandalfus* (Guinot, unpublished data).

**Table 3:** Morphological characters used by McLay (2007).

Species	G2/G1 ratio	G1 setae or spines	Eye mobility	Cornea
*Segonzacia mesatlantica*	G2>G1	setae	mobile	present
*Cyanagraea praedator*	G2≥G1	setae	restricted	present
*Allograea tomentosa*	?	?	mobile	present
*Bythograea thermydron*	G2>G1	setae	mobile	present
*Bythograea galapagensis*	G2>G1	setae	mobile	present
*Bythograea laubieri*	G2>G1	setae	mobile	present
*Bythograea vrijenhoeki*	G2>G1	none	mobile	present
*Bythograea microps*	G2>G1	none	mobile	present
*Austinograea rodriguezensis*	G2<G1	spines	fixed	absent
*Austinograea williamsi*	G2<G1	spines	fixed	absent
*Austinograea alayseae*	G2<G1	spines	fixed	vestigial
*Gandalfus puia*	G2∼G1	spines	fixed	vestigial
*Gandalfus yunohana*	G2∼G1	spines	fixed	present

McLay's [31] suggestion that *Allograea* is basal among bythograeids follows from the observation that this genus seems to be the least modified member of Bythograeidae, because it lacks some modifications that are observed in the other bythograeids [4]. However, our results show that lack of these modifications is not a predictor of the phylogenetic position of this genus. Finally, our results agree with McLay [31] in that *Gandalfus yunohana* belongs to *Gandalfus*; and not to *Austinograea*, where it was originally placed. McLay [31] noted that in *G. yunohana* and *G. puia*, the first male gonopod (G1) is about equal size to the second male gonopod (G2); whereas in the other *Austinograea* species G2 is shorter than G1. Therefore, this character appears to be useful for distinguishing these two sister genera.

### 4.5 Biogeography

Assuming the monophyly of Bythograeidae, our results suggest this family likely arose in the eastern Pacific. An origin in this region is the most parsimonious explanation in any of the three remaining alternative topologies for bythograeid genera (Figure 3). Colonization of the Mid-Atlantic Ridge appears to have occurred early in the diversification of the non-*Bythograea* clade. However, the order of events is unclear given the three alternative positions for the placement of *S. mesatlantica*. Colonization of the other main deep-sea hydrothermal vent regions appears to have followed a stepping-stone progression, as indicated by the phylogenetic relationships and distribution of the monophyletic group *Allograea-Gandalfus-Austinograea*: probably from the East Pacific Rise to the Pacific Antarctic Ridge (*Allograea*), then to the Western Pacific Back-Arc Systems (*Austinograea* and *Gandalfus*), and more recently to the Central Indian Ridge (*A. rodriguezensis*).

The age of Bythograeidae is unknown and fossils for this group are not available. Tudge et al. [25] proposed the Bythograeidae arose during or after the Eocene [a period that occurred 56–34 million years ago (Mya)]. An age <30 My for Bythograeidae might be consistent with their absence on the northeastern Pacific (NEP) ridge systems (i.e., Gorda, Juan de Fuca, and Explorer; Fig. 1). The NEP systems are remnants of the ancestral Pacific-Farallon Ridge, which was located between the Pacific and Farallon plates and extended for ∼10,000 Km [85]. Subduction of the Farallon Plate beneath the North American Plate ∼30 MYA, isolated the NEP ridges from the modern East Pacific Rise (EPR) [86], an event that led to vicariant speciation of several vent annelids [87]. Lack of bythograeids in the NEP system is congruent with an origin along the EPR and/or GAR after the subduction of the Farallon Plate, less than 30 MYA.

A biogeographic pattern is not apparent within the genus *Bythograea*. The distribution of the two *Bythograea* sister species pairs is intriguing, however. Within each sister-species pair, one species occupies a relatively small portion of the range of its respective sister species: *B. galapagensis* in GAR vs. its sister *B. thermydron* in GAR+EPR; and *B. vrijenhoeki* in PAR vs. its sister *B. laubieri* in PAR+Southern EPR. Sympatry of two to four bythograeid species is common at eastern Pacific vent sites, which in some cases include two genera (Figure 1). A longer existence of Bythograeidae in the eastern Pacific may have contributed to the high diversity of genera and species in this region. Sympatric coexistence of several bythograeids in the eastern Pacific suggests niche partitioning during and/or after divergence. In addition, barriers for dispersal may have also contributed to diversification of bythograeids in this region. Deep-sea currents and topographic ridge discontinuities, such as the Easter Microplate that separates the EPR and PAR, may be responsible for the isolation and genetic differentiation observed in vent-endemic organisms restricted to the Galapagos Ridge (GAR) and PAR [88], [89]. Such dispersal barriers may have contributed to the isolation and genetic differentiation of *B. vrijenhoeki* and *A. tomentosa* in PAR and of *B. galapagensis* in GAR.

In contrast to eastern Pacific bythograeids, little overlap is observed in the distributions of *Austinograea* and *Gandalfus* species, suggesting diversification in the Western Pacific (WP) and Central Indian Ridge (CIR) proceeded mainly allopatrically. Both genera probably originated in the WP and discontinuity of Back-Arc basins may have promoted isolation and subsequent differentiation of their populations. Colonization of CIR appears to have occurred early during the diversification of *Austinograea*, as indicated by the basal split between *A. rodriguezensis* (from CIR) and the three *Austinograea* WP lineages. The lack of phylogenetic resolution within WP *Austinograea* could indicate a rapid radiation, however, more genes need to be examined to explore this hypothesis. Each *Gandalfus* species is found at an opposite end of the WP (i.e., *G. puia* in the Kermadec Ridge, New Zealand, and *G. yunohana* off Central Japan), with the WP *Austinograea* species found in the range between them.

The diversity of bythograeids observed in Pacific vent systems is in striking contrast to the single species reported in the CIR and Mid-Atlantic Ridge (MAR). It is possible that other species in addition to *A. rodriguezensis* are present in CIR, because this region has not been sufficiently explored. However, the MAR has been extensively surveyed and only *S. mesatlantica* has been found. Preliminary examination of the mitochondrial Cytb gene revealed little genetic variation throughout the range of *S. mesatlantica* (Hurtado, unpublished data). High dispersal ability and/or lack of effective dispersal barriers across its known range may have prevented allopatric differentiation. In addition, it is possible that the more stable communities of the long-lived hydrothermal vents of MAR provide fewer opportunities for bythograeid diversification than the ephemeral vent communities of the Eastern Pacific. The existence of different succession stages in the eastern Pacific vent communities may have provided opportunities for Bythograeidae lineages to adapt to, and appear at different stages. Although *B. thermydron* is present during multiple succession stages of the vent communities [20], some of the rarely found species may be more specific to certain succession stages, but this remains to be determined.

### 4.6 Conclusions

Our study illustrates how three Bayesian species tree inference methods differ in the way in which they weigh information in genes to estimate a species tree. Differences in posterior probabilities of certain clades were observed between methods, despite apparently small differences in assumptions and implementation (i.e., BEST and *Beast). This is particularly relevant as most current multilocus phylogenetic studies use at most one of these methods. Incongruent results among species tree methods in our study and in the limited published reports that have compared these methods with empirical data, caution against the use of a single species tree method. Further comparison of these methods with empirical and simulated data is needed to better understand the nature of these incongruences.

Our study resolved some of the relationships within the family Bythograeidae, and refutes some of the relationships previously proposed on the basis of morphology. It also allowed for several inferences about the biogeography of this group. Finally, although our outgroup analyses were largely inconclusive, they indicate that *Calocarcinus* does not represent the closest relative of bythograeids.

## Supporting Information

Figure S1 **Results of “Outgroup Identification” analyses.** Majority-rule consensus trees of RaxML bootstrap analyses from four datasets. A. 28S rDNA gene. B. Mitochondrial (16S rDNA, COI, Cyt b). C. 16S rDNA and H3A. D. Nak. Bolded taxon labels represent the family Bythograeidae. Numbers to the left of a node are % bootstrap support. Aligned datasets, including GenBank accession numbers for previously published sequences, are available in the Supporting Information Datasets S1, S2, S3, S4, S5.
(TIF)

Table S1 **Description of datasets used for the Outgroup Identification phylogenetic analyses based on multiple Brachyuran taxa.** Corresponding best-fit models according to the Akaike Information Criterion (AIC), the corrected AIC (AICc), and the Bayesian Information Criterion (BIC) are shown.
(DOC)

Table S2 **Parameters assumed for each analysis conducted for the ingroup taxa (Family Bythograeidae only).**
(DOC)

Table S3 **Bootstrap or Posterior probability support for three clades in the Bythograeidae family.** Based on analyses of multiple Brachyuran taxa. Empty cells indicate less than 50% clade support. Alternative relationships were not supported.
(DOC)

Table S4 **Clade support (Maximum Likelihood Bootstrap proportions, Bayesian Posterior Probabilities, and Concordance Factors)**. Measures of clade support obtained for each of the methods, datasets, and assumptions examined. Clade names correspond to those depicted in Figure 2. Empty cells represent clades that received <50% support in corresponding analysis.
(DOC)

Table S5 **Percent divergences among members of the genus** ***Bythograea*** **and among genera in the family Bythograeidae.** Divergences are Kimura-2-Parameter-corrected distances. Above diagonal: based on three nuclear genes combined (28S rDNA, NaK, and H3A). Below diagonal: based on three mitochondrial genes combined (16S rDNA, COI, and Cytb).
(DOC)

Dataset S1 **Sequence alignment of 16S rDNA, COI, and Cytb genes used in the Outgroup Identification analyses.** (20 taxa; Nexus format).
(NEX)

Dataset S2 **Sequence alignment of 16S rDNA and H3A genes used in the Outgroup Identification analyses.** (28 taxa; Nexus format).
(NEX)

Dataset S3 **Sequence alignment of the 28S rDNA gene used in the Outgroup Identification analyses.** (23 taxa; Nexus format).
(NEX)

Dataset S4 **Sequence alignment of the NaK gene used in the Outgroup Identification analyses.** (23 taxa; Nexus format).
(NEX)

Dataset S5 **Sequence alignment of the H3A gene used in the Outgroup Identification analyses.** (284 taxa; Nexus format).
(NEX)

Dataset S6 **Sequence alignment of the 16S rDNA gene used in the ingroup analyses.** (13 taxa; Nexus format).
(NEX)

Dataset S7 **Sequence alignment of the 28S rDNA gene used in the ingroup analyses.** (13 taxa; Nexus format).
(NEX)

Dataset S8 **Sequence alignment of the 16S rDNA, COI, Cytb, 28S rDNA, H3A, and NaK genes used in the ingroup analyses.** (10 taxa; Nexus format).
(NEX)
