# Supplementary figures and images for: Molecular Systematics of the Deep-Sea Hydrothermal Vent Endemic Brachyuran Family Bythograeidae: A Comparison of Three Bayesian Species Tree Methods
Source: PLoS One. 2012 Mar 5;7(3):e32066. doi: 10.1371/journal.pone.0032066 (PMC3293879; doi:10.1371/journal.pone.0032066)

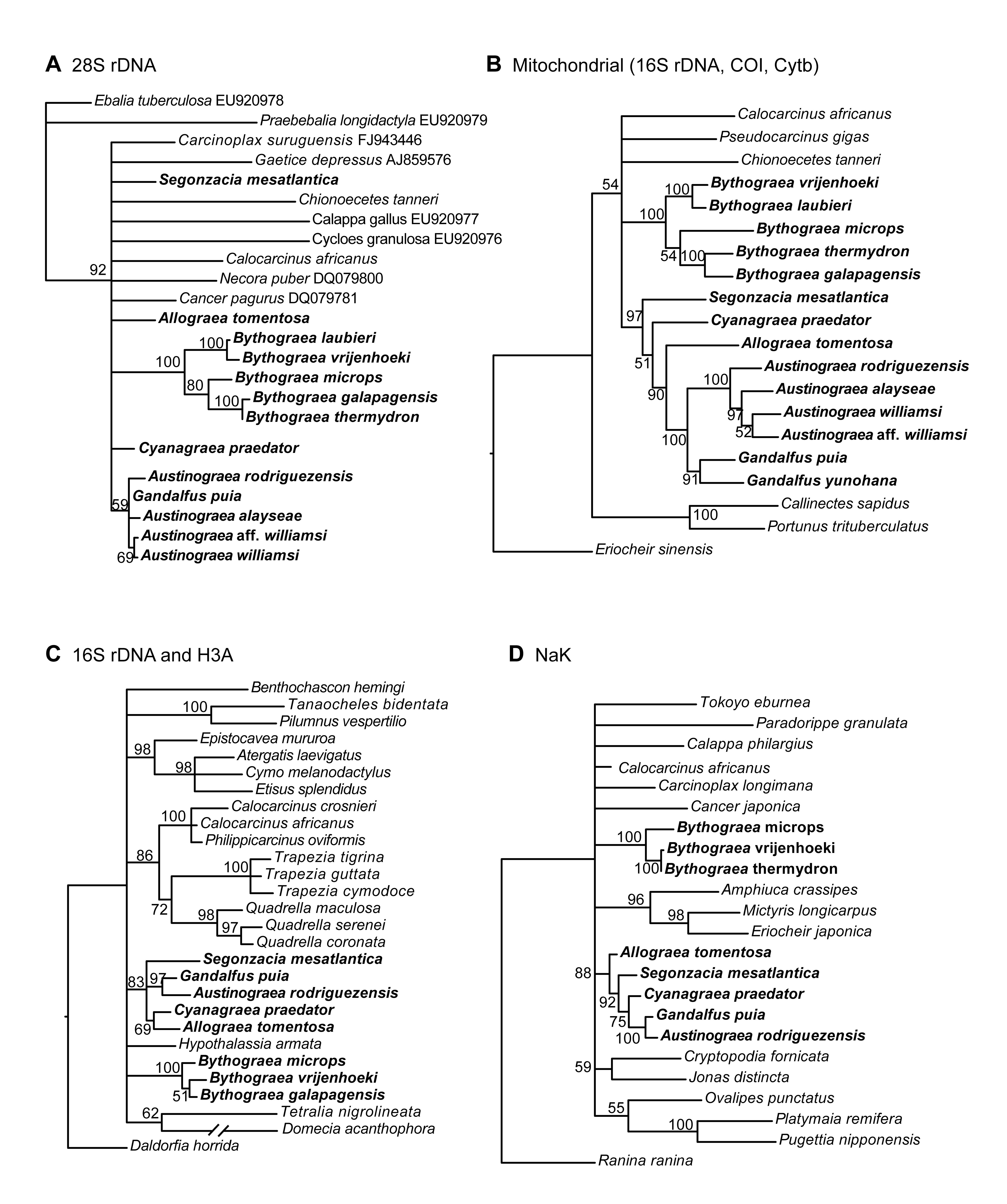

Supplement: Figure S1 — Results of “Outgroup Identification” analyses. Majority-rule consensus trees of RaxML bootstrap analyses from four datasets. A. 28S rDNA gene. B. Mitochondrial (16S rDNA, COI, Cyt b). C. 16S rDNA and H3A. D. Nak. Bolded taxon labels represent the family Bythograeidae. Numbers to the left of a node are % bootstrap support. Aligned datasets, including GenBank accession numbers for previously published sequences, are available in the Supporting Information Datasets S1, S2, S3, S4, S5. (TIF) [file pone.0032066.s001.tif]
