# Supplementary material for: Molecular Systematics of the Deep-Sea Hydrothermal Vent Endemic Brachyuran Family Bythograeidae: A Comparison of Three Bayesian Species Tree Methods
Source: PLoS One. 2012 Mar 5;7(3):e32066. doi: 10.1371/journal.pone.0032066 (PMC3293879; doi:10.1371/journal.pone.0032066)
Supplement: Table S1 — Description of datasets used for the Outgroup Identification phylogenetic analyses based on multiple Brachyuran taxa. Corresponding best-fit models according to the Akaike Information Criterion (AIC), the corrected AIC (AICc), and the Bayesian Information Criterion (BIC) are shown. (DOC) [file pone.0032066.s002.doc]

| Genes Examined | No. Taxa | Outgroup(s) | No. excluded characters | No. retained characters | | No. parsimony informative characters | | AIC (weight) | | AICc (weight) | | BIC (weight) |
| --- | --- | --- | --- | --- | --- | --- | --- | --- | --- | --- | --- | --- |
| 28S | 23 | *Praebebalia longidactyla* | 84 | 608 | | 85 | | GTR+I+G (0.97) | | GTR+I+G (0.95) | | TIM3+I+G (0.66) |
| NaK | 23 | *Ranina ranina* | 0 | 523 | | 159 | | TIM2+I+G (0.78) | | TIM2+I+G (0.78) | | TIM2ef+I+G (0.84) |
| H3A | 284 | *Pachygrapsus marmoratus* | 0 | 328 | 134 | | TIM3+I+G (0.49) | | TIM3+I+G (0.42) | | TPM3uf+I+G (0.30) | |
| 16S H3A | 28 | *Daldorfia horrida* | 239 | 644 | | 157 | | SYM+I+G (0.74) | | SYM+I+G (0.85) | | SYM+I+G (0.98) |
| 16S COI Cytb | 20 | *Eriocheir sinensis; Callinectes sapidus*; *Portunus trituberculatus* | 90 | 1471 | | 491 | | TIM2+I+G (0.44) | | TIM2+I+G (0.45) | | HKY+I+G (0.60) |
