# Supplementary material for: Molecular Systematics of the Deep-Sea Hydrothermal Vent Endemic Brachyuran Family Bythograeidae: A Comparison of Three Bayesian Species Tree Methods
Source: PLoS One. 2012 Mar 5;7(3):e32066. doi: 10.1371/journal.pone.0032066 (PMC3293879; doi:10.1371/journal.pone.0032066)
Supplement: Table S2 — Parameters assumed for each analysis conducted for the ingroup taxa (Family Bythograeidae only). (DOC) [file pone.0032066.s003.doc]

| Method and Partition | Model and priors | Bootstrap replicates or MCMC length (sampling frequency) |
| --- | --- | --- |
| Concatenated (6 genes 10 taxa): |  |  |
| RaxML one partition | GTR G | 1000 |
| RaxML partition by gene | GTR G | 1000 |
| RaxML partition by linkage group | GTR G | 1000 |
| GARLI one partition | GTR G | 1000 |
| MrBayes one partition | GTR G (nst=6 rates=gamma) | 300–400 million x 4 runs (10,000) |
| BayesPhylogenies (1–6 partitions) | GTR G | 200–500 million x 8 runs (5,000) |
|  |  |  |
| Species tree: |  |  |
| *Beast (10 taxa and 6 taxa) |  | 1000 million (100,000) |
| 28S | GTR G |  |
| mitochondrial (16S COI Cytb) | GTR G |  |
| H3A | HKY |  |
| Nak | HKY G |  |
|  |  |  |
| BEST (6 taxa) |  | 200 million x 4 runs (5,000) |
| 28S | GTR G (nst=6 rates=gamma ploidy=diploid) |  |
| mitochondrial (16S COI Cytb) | GTR G (nst=6 rates=gamma ploidy=haploid) |  |
| H3A | HKY (nst=2 rates=equal ploidy=diploid) |  |
| Nak | HKY G (nst=2 rates=gamma ploidy=diploid) |  |
| coalescent parameters | prset applyto=(all) thetapr=invgamma(3,0.03) GeneMuPr=uniform(0.5,1.5) best=1 |  |
| unlinked parameters | unlink topology=(all) brlens=(all) statefreq=(all) genemu=(all) revmat=(all) shape=(all) |  |
|  |  |  |
| Bayesian Concordance Analyses |  |  |
| 28S | GTR G MrBayes (nst=6 rates=gamma) | 500 million x 8 runs |
| mitochondrial (16S COI Cytb) | GTR G MrBayes (nst=6 rates=gamma) | 500 million x 8 runs |
| H3A | HKY MrBayes (nst=2 rates=equal) | 500 million x 8 runs |
| Nak | HKY G MrBayes (nst=2 rates=equal) | 500 million x 8 runs |
|  |  |  |
| BUCKy | alfa priors tested: 0.01, 0.5, 1, 2, 5, 10, 10000 | 10 million |
