# Supplementary material for: Molecular Systematics of the Deep-Sea Hydrothermal Vent Endemic Brachyuran Family Bythograeidae: A Comparison of Three Bayesian Species Tree Methods
Source: PLoS One. 2012 Mar 5;7(3):e32066. doi: 10.1371/journal.pone.0032066 (PMC3293879; doi:10.1371/journal.pone.0032066)
Supplement: Table S3 — Bootstrap or Posterior probability support for three clades in the Bythograeidae family. Based on analyses of multiple Brachyuran taxa. Empty cells indicate less than 50% clade support. Alternative relationships were not supported. (DOC) [file pone.0032066.s004.doc]

| Genes examined | Method | Model | Genus *Bythograea* | *GAASC*^a^ | *Gandalfus-Austinograea* |
| --- | --- | --- | --- | --- | --- |
| 28S rDNA | RaxML | GTR G | 100 |  | 62 |
|  | Garli | GTR G | 99 |  |  |
|  | Garli | TIM3 G | 99 |  |  |
|  | MrBayes | GTR G | 100 |  | 99 |
|  |  |  |  |  |  |
| Nak | RaxML | GTR G | 100 | 89 | 100 |
|  | Garli | GTR G | 99 | 89 | 99 |
|  | Garli | TIM2 G | 99 | 82 | 99 |
|  | Garli | GTR G | 100 | 79 | 99 |
|  | MrBayes | GTR G | 100 | 100 | 100 |
|  |  |  |  |  |  |
| H3A | RaxML | GTR G | 74 | 65 | 90 |
|  | Garli | GTR G | 70 |  | 80 |
|  | Garli | TPM3uf G | 73 |  | 79 |
|  | Garli | TIM3 G | 73 | 50 | 81 |
|  |  |  |  |  |  |
| 16S rDNA, H3A | RaxML | GTR G | 100 | 83 | 99 |
|  | Garli | GTR G | 100 | 61 | 100 |
|  | Garli | SYM G | 100 | 64 | 88 |
|  | MrBayes | GTR G | 100 | 90 | 100 |
|  |  |  |  |  |  |
| 16S rDNA, COI, Cytb | RaxML | GTR G | 100 | 98 | 99 |
|  | Garli | GTR G | 100 | 88 | 98 |
|  | MrBayes | GTR G | 100 | 100 | 94 |

^a^*Gandalfus-Austinograea-Allograea-Segonzacia-Cyanagraea*
