# Supplementary material for: Molecular Systematics of the Deep-Sea Hydrothermal Vent Endemic Brachyuran Family Bythograeidae: A Comparison of Three Bayesian Species Tree Methods
Source: PLoS One. 2012 Mar 5;7(3):e32066. doi: 10.1371/journal.pone.0032066 (PMC3293879; doi:10.1371/journal.pone.0032066)
Supplement: Table S4 — Clade support (Maximum Likelihood Bootstrap proportions, Bayesian Posterior Probabilities, and Concordance Factors). Measures of clade support obtained for each of the methods, datasets, and assumptions examined. Clade names correspond to those depicted in Figure 2. Empty cells represent clades that received <50% support in corresponding analysis. (DOC) [file pone.0032066.s005.doc]

|  |  | |  | |  | | *GAASC* Clade | | | | | | | | | | | | | | | | | | | | | Genus *Bythograea* | | | | | | | | | | | | |
| --- | --- | --- | --- | --- | --- | --- | --- | --- | --- | --- | --- | --- | --- | --- | --- | --- | --- | --- | --- | --- | --- | --- | --- | --- | --- | --- | --- | --- | --- | --- | --- | --- | --- | --- | --- | --- | --- | --- | --- | --- |
| **Method** | **Taxon Set^a^** | | **Assumptions^b^** | | **Marginal Posterior Prob.^c^ (–lnL)** | | **Clade A**: *G-Au* | | | **Clade B**: *G-Au-Al* | | | *G-Au-Al-C* | | | *S-C* | | | | *G-Au-Al-S* | | | *G-Au-S-C* | | | *G-Au-C* | | **Clade C**: *T-G* | | | **Clade E**: *V-L* | | | *V-L-M* | | | *T-G-V-L* | | |  |
|  | | | | | | | | | | | | | | | | | | | | | | | | | | | | | | | | | | | | | | | | |
| **A. Four linkage groups (28S, mitochond, H3A, NaK)** | | | | | | | | | | | | | | | | | | | | | | | | | | | | | | | | | | | | | | | | |
| Garli | 10 | | 1 |  | | 100 | | | 97 | | |  | | | 53 | | |  | | | |  | | |  | | 100 | | | 100 | | 60 | | | |  | |  | | |
| RaxML | 10 | | 1 |  | | 100 | | | 99 | | |  | | |  | | |  | | | |  | | |  | | 100 | | | 100 | | 55 | | | |  | |  | | |
| RaxML | 10 | | 4 linkage gps. | | | 100 | | | 100 | | | 52 | | |  | | |  | | | |  | | |  | | 100 | | | 100 | | 68 | | | |  | |  | | |
| RaxML | 10 | | 6 gene | | | 100 | | | 100 | | | 66 | | |  | | |  | | | |  | | |  | | 100 | | | 100 | | 56 | | | |  | |  | | |
| MrBayes | 10 | | 1 | 10195.98 | | 100 | | | 100 | | |  | | | 62 | | |  | | | |  | | |  | | 100 | | | 100 | | 73 | | | |  | |  | | |
| BP | 10 | | 1 | 10195.61 | | 100 | | | 100 | | |  | | | 63 | | |  | | | |  | | |  | | 100 | | | 100 | | 71 | | | |  | |  | | |
| BP | 10 | | 2 | 10100.16 | | 100 | | | 100 | | |  | | |  | | |  | | | |  | | |  | | 100 | | | 100 | | 60 | | | |  | |  | | |
| BP | 10 | | 3 | 10062.98 | | 100 | | | 100 | | |  | | |  | | |  | | | |  | | |  | | 100 | | | 100 | |  | | | |  | |  | | |
| BP | 10 | | 4 | **10052.39** | | 100 | | | 100 | | |  | | |  | | |  | | | |  | | |  | | 100 | | | 100 | |  | | | | 54 | |  | | |
| BP | 10 | | 5 | 10054.72 | | 100 | | | 100 | | |  | | |  | | |  | | | |  | | |  | | 100 | | | 100 | |  | | | |  | |  | | |
| BP | 10 | | 6 | 10054.57 | | 100 | | | 100 | | |  | | |  | | |  | | | |  | | |  | | 100 | | | 100 | |  | | | |  | |  | | |
| *BEAST | 10 | | 4 | 9522.66 | | 100 | | | 78 | | |  | | | 90 | | |  | | | |  | | |  | | 100 | | | 100 | |  | | | | 88 | |  | | |
| *BEAST | 6 *GAASC* | | 4 | 7979.71 | | 100 | | | 68 | | |  | | | 82 | | |  | | | |  | | |  | | na | | na | | | na | | | | na | |  | | |
| *BEAST | 6 *Bytho.* | | 4 | NC | | na | | | na | | | na | | | na | | | na | | | | na | | | na | | na | | na | | | na | | | | na | |  | | |
| BEST | 6 *GAASC* | | 4 | 8416.12 | | 100 | | |  | | |  | | |  | | |  | | | |  | | |  | | na | | na | | | na | | | | na | |  | | |
| BEST | 6 *Bytho.* | | 4 | 7446.35 | | na | | | na | | | na | | | na | | |  | | | |  | | |  | | 93 | | 100 | | | 54 | | | |  | |  | | |
|  | | | | | | | | | | | | | | | | | | | | | | | | | | | | | | | | | | | | | | | | |
| **B. Three linkage groups- No Nak (28S rDNA, mt, H3A)** | | | | | | | | | | | | | | | | | | | | | | | | | | | | | | | | | | | | | | | | |
| *BEAST | 10 | | 3 | 7951.25 | | 99 | | 85 | | |  | | | 94 | | |  | | | |  | | |  | | | 100 | | | 100 | | |  | | | 69 | | |  | |
| *BEAST | 6 *GAASC* | | 3 | 8242.51 | | 98 | | 80 | | |  | | | 92 | | |  | | | |  | | |  | | | na | | | na | | | na | | | na | | |  | |
| *BEAST | 6 *Bytho.* | | 3 | NC | | na | | na | | | na | | | na | | | na | | | | na | | | na | | | na | | | na | | | na | | | na | | |  | |
| BEST | 6 *GAASC* | | 3 | 6713.87 | | 98 | | 99 | | |  | | |  | | | 63 | | | |  | | |  | | | na | | | na | | | na | | | na | | |  | |
| BEST | 6 *Bytho.* | | 3 | 6058.70 | | na | | na | | | na | | | na | | | na | | | | na | | | na | | | 100 | | | 100 | | | 51 | | |  | | |  | |
|  | | | | | | | | | | | | | | | | | | | | | | | | | | | | | | | | | | | | | | | | |
| **C. Three linkage groups- No H3A (28S rDNA, mt, NaK)** | | | | | | | | | | | | | | | | | | | | | | | | | | | | | | | | | | | | | | | | |
| *BEAST | 10 | | 3 | 8882.81 | | 99 | | | 76 | | |  | | | 88 | | | |  | | |  | | |  | | 99 | | 99 | | |  | | | | 89 | |  | | |
| BEST | 6 *GAASC* | | 3 | 7545.61 | | 100 | | | 78 | | |  | | |  | | | |  | | |  | | |  | | na | | na | | | na | | | | na | |  | | |
| BEST | *6 Bytho.* | | 3 | 6857.46 | | na | | | na | | | na | | | na | | | | na | | | na | | | na | | 99 | | 100 | | | 83 | | | |  | |  | | |
|  | | | | | | | | | | | | | | | | | | | | | | | | | | | | | | | | | | | | | | | | |
| **D. Three linkage groups- No 28S rDNA (mt, H3A, NaK)** | | | | | | | | | | | | | | | | | | | | | | | | | | | | | | | | | | | | | | | | |
| *BEAST | 10 | | 3 | 8547.46 | | 97 | | | 76 | | |  | | | 80 | | | |  | | |  | | |  | | 99 | | 100 | | |  | | | | 92 | |  | | |
| BEST | 6 *GAASC* | | 3 | 7067.45 | | 100 | | | 98 | | |  | | |  | | | | 52 | | |  | | |  | | na | | na | | | na | | | | na | |  | | |
| BEST | *6 Bytho.* | | 3 | 6318.43 | | na | | | na | | | na | | | na | | | | na | | | na | | | na | | 82 | | 99 | | |  | | | |  | |  | | |
|  | | | | | | | | | | | | | | | | | | | | | | | | | | | | | | | | | | | | | | | | |
| **E. Three linkage groups- No mt (28S rDNA, H3A, NaK)** | | | | | | | | | | | | | | | | | | | | | | | | | | | | | | | | | | | | | | | | |
| *BEAST | 10 | | 3 | 3570.82 | | 100 | | |  | | | 62 | | |  | | | |  | | |  | | |  | | 100 | | 100 | | |  | | | | 90 | |  | | |
| BEST | 6 *GAASC* | | 3 | 3270.84 | | 100 | | |  | | | 60 | | |  | | | |  | | |  | | |  | | na | | na | | | na | | | | na | |  | | |
| BEST | *6 Bytho.* | | 3 | 3044.10 | | na | | | na | | | na | | | na | | | | na | | | na | | | na | | 98 | | 100 | | |  | | | | 64 | |  | | |
|  | | | | | | | | | | | | | | | | | | | | | | | | | | | | | | | | | | | | | | | | |
| **F. Individual linkage groups** | | | | | | | | | | | | | | | | | | | | | | | | | | | | | | | | | | | | | | | | |
| MrBayes | 10; 28S | |  | 1288.98 | | 98 | | |  | | |  | | | 70 | | | |  | | | 72 | | |  | | 85 | | | 100 | | 60 | | | |  | |  | | |
| MrBayes | 10; mt | |  | 6266.42 | | 100 | | | 96 | | |  | | | 67 | | | |  | | |  | | |  | | 100 | | | 100 | | 64 | | | |  | |  | | |
| MrBayes | 10; H3A | |  | 689.25 | | 100 | | |  | | | 68 | | |  | | | |  | | |  | | |  | | 99 | | | 96 | |  | | | |  | |  | | |
| MrBayes | 10; NaK | |  | 1597.82 | | 100 | | |  | | | 63 | | |  | | | |  | | |  | | | 75 | | 51 | | | 96 | |  | | | | 82 | |  | | |
|  | | | | | | | | | | | | | | | | | | | | | | | | | | | | | | | | | | | | | | | | |
| **G. Bayesian Concordance Analysis** | | | | | | | | | | | | | | | | | | | | | | | | | | | | | | | | | | | | | | | | |
| BUCKy | 10 | | α = 0.01 | | | 100 | | | 100 | | | 76 | | | 21 | | | |  | | |  | | |  | | 100 | | | 100 | | 51 | | | 48 | | |  | | |
| BUCKy | 10 | | α = 0.5 | | | 100 | | | 100 | | | 76 | | | 21 | | | |  | | |  | | |  | | 100 | | | 100 | | 51 | | | 47 | | |  | | |
| BUCKy | 10 | | α = 1 | | | 100 | | | 100 | | | 76 | | | 21 | | | |  | | |  | | |  | | 100 | | | 100 | | 50 | | | 47 | | |  | | |
| BUCKy | 10 | | α =10 | | | 100 | | | 99 | | | 76 | | | 21 | | | |  | | |  | | |  | | 100 | | | 100 | | 51 | | | 48 | | |  | | |
| BUCKy | | 10 | α =1000 | | | 100 | | | 94 | | | 71 | | | 25 | | | |  | | |  | | |  | | 99 | | | 100 | | 50 | | | 47 | | |  | | |

^a^ BEST only allows one outgroup taxon. *GAASC* dataset = the five members of the *GAASC* clade plus *Bythograea thermydron* as the outgroup. *Bytho.* = the five members of the genus *Bythograea* plus *Gandalfus puia* as the outgroup.

^b^ numbers represent number of partitions. Unless otherwise noted, these correspond to the linkage groups (except for BayesPhylogenies); α = a priori level of discordance among linkage groups.

^c^ Bold is the best of six BayesPhylogenies partitioning schemes according to Bayes factors (see text).

*G = Gandalus; Au = Austinograea; Al = Allograea; S = Segonzacia; C = Cyanagraea*; *T = Bythograea thermydron; G = B. galapagensis; V = B. vrijenhoeki; L = B. laubieri; M = B. microps.*

mt = mitochondrial genes (16S rDNA, COI, Cytb).

na = not applicable because corresponding taxa were not examined.

BP = BayesPhylogenies.

NC = no convergence on a stationary distribution was achieved; thus, results are not reported.
