# Supplementary material for: Molecular Systematics of the Deep-Sea Hydrothermal Vent Endemic Brachyuran Family Bythograeidae: A Comparison of Three Bayesian Species Tree Methods
Source: PLoS One. 2012 Mar 5;7(3):e32066. doi: 10.1371/journal.pone.0032066 (PMC3293879; doi:10.1371/journal.pone.0032066)
Supplement: Table S5 — Percent divergences among members of the genus Bythograea and among genera in the family Bythograeidae. Divergences are Kimura-2-Parameter-corrected distances. Above diagonal: based on three nuclear genes combined (28S rDNA, NaK, and H3A). Below diagonal: based on three mitochondrial genes combined (16S rDNA, COI, and Cytb). (DOC) [file pone.0032066.s006.doc]

|  | *B. thermydron* | *B. galapagensis* | *B. laubieri* | *B. vrijenjoeki* | *B. microps* | *G. puia* | *Au. rodriguezensis* | *Al. tomentosa* | *S. mesatlantica* | *C. praedator* |
| --- | --- | --- | --- | --- | --- | --- | --- | --- | --- | --- |
| *B. thermydron* |  | 0.12 | 1.21 | 1.22 | 1.84 | 6.00 | 6.33 | 5.72 | 5.93 | 5.36 |
| *B. galapagensis* | 7.65 |  | 1.27 | 1.28 | 1.96 | 6.14 | 6.47 | 5.86 | 6.07 | 5.49 |
| *B. laubieri* | 10.90 | 12.02 |  | 0.24 | 2.15 | 6.24 | 6.44 | 6.12 | 6.04 | 5.72 |
| *B. vrijenjoeki* | 11.32 | 12.45 | 4.36 |  | 2.41 | 6.43 | 6.64 | 6.18 | 6.23 | 5.91 |
| *B. microps* | 13.62 | 13.22 | 13.77 | 13.88 |  | 6.69 | 6.97 | 6.18 | 6.36 | 5.97 |
| *G. puia* | 17.23 | 17.70 | 15.89 | 16.07 | 18.41 |  | 1.50 | 2.69 | 2.92 | 2.18 |
| *Au. rodriguezensis* | 18.99 | 18.63 | 17.27 | 18.18 | 19.93 | 13.84 |  | 3.16 | 3.23 | 2.62 |
| *Al. tomentosa* | 18.97 | 19.05 | 18.20 | 18.69 | 20.15 | 13.61 | 15.06 |  | 2.68 | 1.76 |
| *S. mesatlantica* | 19.30 | 17.88 | 16.96 | 17.51 | 19.47 | 14.97 | 16.21 | 15.73 |  | 2.18 |
| *C. praedator* | 17.09 | 16.98 | 17.51 | 17.63 | 19.51 | 14.02 | 14.62 | 13.26 | 13.39 |  |
